# Aging aggravated liver ischemia and reperfusion injury by promoting STING‐mediated NLRP3 activation in macrophages

**DOI:** 10.1111/acel.13186

**Published:** 2020-07-14

**Authors:** Weizhe Zhong, Zhuqing Rao, Jianhua Rao, Guoyong Han, Ping Wang, Tao Jiang, Xiongxiong Pan, Shun Zhou, Haoming Zhou, Xuehao Wang

**Affiliations:** ^1^ Hepatobiliary/Liver Transplantation Center The First Affiliated Hospital with Nanjing Medical University Nanjing China; ^2^ Research Unit of Liver Transplantation and Transplant Immunology Chinese Academy of Medical Sciences Nanjing China; ^3^ Key Laboratory of Liver Transplantation Chinese Academy of Medical Sciences Nanjing China; ^4^ Department of Anesthesiology The First Affiliated Hospital with Nanjing Medical University Nanjing China

**Keywords:** aging, leucine‐rich repeat containing protein 3, liver ischemia, and reperfusion injury, macrophage immune response, nucleotide‐binding domain, stimulator of interferon genes

## Abstract

Although aggravated liver injury has been reported in aged livers post‐ischemia and reperfusion (IR), the underlying mechanism of innate immune activation of aged macrophages is not well understood. Here, we investigated whether and how Stimulator of interferon genes (STING) signaling regulated macrophage proinflammatory activation and liver IR injury. Mice were subjected to hepatic IR in vivo. Macrophages isolated from IR‐stressed livers and bone marrow‐derived macrophages (BMDMs) from young and aged mice were used for in vitro studies. Enhanced nucleotide‐binding domain and leucine‐rich repeat containing protein 3 (NLRP3) activation was found in both livers and macrophages of aged mice post‐IR. NLRP3 knockdown in macrophages inhibited intrahepatic inflammation and liver injury in both young and aged mice. Interestingly, enhanced activation of the STING/ TANK‐binding kinase 1 (TBK1) signaling pathway was observed in aged macrophages post‐IR and mitochondria DNA (mtDNA) stimulation. STING suppression blocked over‐activation of NLRP3 signaling and excessive secretion of proinflammatory cytokines/chemokines in the mtDNA‐stimulated BMDMs from aged mice. More importantly, STING knockdown in macrophages abrogated the detrimental role of aging in aggravating liver IR injury and intrahepatic inflammation. Finally, peripheral blood from the recipients undergoing liver transplantation was collected and analyzed. The results showed that the elderly recipients had much higher levels of TNF‐α, IL‐6, IL‐1β, and IL‐18 post‐transplantation, indicating increased NLRP3 activation in lR‐stressed livers of elderly recipients. In summary, our study demonstrated that the STING‐NLRP3 axis was critical for the proinflammatory response of aged macrophages and would be a novel therapeutic target to reduce IR injury in elderly patients.

## Graphical abstract

Liver IR triggered mtDNA release from stressed hepatocytes. Aging promoted STING over‐activation, leading to enhanced NLRP3 inflammasome activation and increased proinflammatory cytokines/chemokines production of macrophages, which ultimately aggravated IR injury.

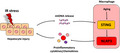

## 1 INTRODUCTION

With an increasingly aging population, more elderly patients are likely to develop hepatic malignancies that are amenable to liver surgeries. IR injury is a multifactorial process that affects liver function post–liver partial resection and transplantation (Zhai, Petrowsky, Hong, Busuttil, & Kupiec‐Weglinski, 2013). Increased sensitivity of the aged liver to IR injury has been reported (Chun et al., 2018). However, protective strategies are still lacking and need to be further studied.

The inflammatory response is an important factor that contributes to the aging progress as well as hepatic IR injury (Kan, Ungelenk, Lupp, Dirsch, & Dahmen, 2018). Macrophages play a critical role in the pathogenesis of liver IR injury (Lu et al., 2016). Activation of macrophages in response to pathogen‐associated molecular patterns (PAMPs) or damage‐associated molecular patterns (DAMPs) enhances the recruitment and activation of other innate and adaptive immune cells to amplify the intrahepatic inflammation.

NLRP3 is a well‐studied inflammasome that induces strong proinflammatory responses upon activation. NLRP3 activation in macrophages has been shown to promote inflammation and hepatocellular injury in livers post‐IR (Mohamadi et al., 2018). Endogenous extracellular histones activate the NLRP3 inflammasome in Kupffer cells (KCs) induced sterile inflammatory live IR injury (Huang et al., 2013). Reactive oxygen species (ROS)‐mediated activation of the NLRP3 and absent in melanoma 2 (AIM2) inflammasomes in KCs were also found to promote IR‐induced inflammatory responses (Kim, Kim, & Lee, 2015). Additionally, autophagy blockade led to the accumulation of ROS‐generating mitochondria, which further activated the NLRP3 inflammasome (Zhou, Yazdi, Menu, & Tschopp, 2011). In aged mice, spontaneously elevated systemic levels of TNF activated the NLRP3 inflammasome in liver and adipose tissues (Bauernfeind, Niepmann, Knolle, & Hornung, 2016). However, little is known about the role of NLRP3 inflammasome activation during IR in aged mice.

STING is a universal receptor that recognizes released DNA and triggers innate immune activation, which has important functions in infection, inflammation and cancer (Barber, 2015). In liver, STING‐mediated inflammation in macrophages contributed to the progression of non‐alcoholic steatohepatitis in both humans and mice (Yu et al., 2019; Luo et al., 2018). Lack of immunological DNA sensing in hepatocytes facilitated hepatitis B virus (HBV) infection, and introduction of STING expression specifically in hepatocytes reconstituted the DNA‐sensing pathway, leading to improved control of HBV infection (Thomsen et al., 2016). Few data were available about the role of STING signaling in liver IR injury. A recent study showed that there was no significant difference in liver IR injury between WT and STING‐deficient mice (Lei et al., 2018). Interplay of STING and NLRP3 has been recently revealed. In an LPS‐induced cardiac injury model, STING activation by LPS stimulation triggered ROS‐dependent NLRP3 activation, and NLRP3 overexpression by adenovirus abrogated the protective effects of STING knockdown in LPS‐induced cardiomyocytes (Li, Zhou, et al., 2019). However, whether STING signaling affected NLRP3 inflammasome activation and liver injury in aged mice post‐IR remains unclear.

In the present study, we investigated whether and how STING signaling regulated liver IR injury in aged mice. We demonstrated that liver IR triggered over‐activation of the NLRP3 inflammasome in macrophages in a STING‐dependent manner, which contributed to the increased intrahepatic inflammation and liver injury in the aged mice.

## 2 RESULTS

### 2.1 Aging aggravated hepatocellular injury and intrahepatic inflammation in IR‐stressed livers

**FIGURE 1:**
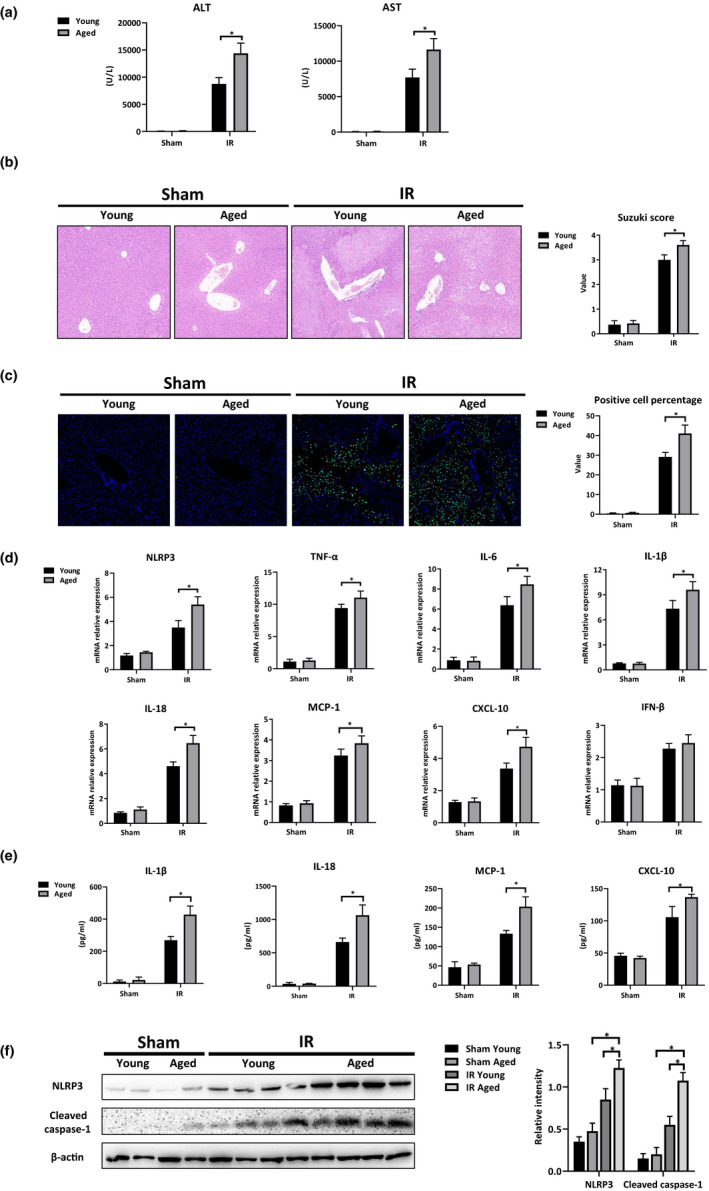
Aging aggravated hepatocellular injury and intrahepatic inflammation in IR‐stressed livers. Young and aged mice were subjected to liver partial warm ischemia for 1.5 hr followed by 6 hr of reperfusion. (a) Average levels of serum ALT and AST in mice. (b) H&E‐stained tissue sections of livers; Suzuki scores were based on liver H&E‐stained sections. (c) TUNEL‐stained sections of liver tissues; Positive cell percentage was evaluated by ImageJ software. (d) Inflammation‐related gene expressions (NLRP3, TNF‐α, IL‐6, IL‐1β, IL‐18, MCP‐1, CXCL‐10, and IFN‐β) were measured by qRT‐PCR; Average target gene/GAPDH ratios of the different experimental groups were presented. (e) IL‐1β/IL‐18/MCP‐1/CXCL‐10 in mice serum measured by ELISA. (f) Protein lysates were prepared from liver tissues and subjected to Western blot analysis, which was used to determine the levels of NLRP3, Cleaved caspase‐1, and β‐actin expressions. Relative intensity was analyzed by ImageJ software. n = 6 mice/group. All results were representative of at least two independent experiments. Values were presented as the mean ± *SD*. Significance (*p*‐value) was determined by *t* test, *p < 0.05.

First, we sought to determine whether aging increased liver IR injury. The young and aged mice were subjected to IR or the sham procedure. After 6 hr of reperfusion, the extent of the liver injury and intrahepatic inflammation was compared between the groups. Compared with the levels observed in the young group, the aged group showed significantly higher levels of serum ALT and AST (Figure 1a), fewer preserved liver architectures, higher Suzuki scores (Figure 1b) and more TUNEL‐positive stained hepatocytes (Figure 1c), which indicated exacerbated liver injury.

NLRP3, a danger signal sensor, is essential for the initiation of profound, sterile inflammation during liver IR injury (Li, Jin, et al., 2019; Xu et al., 2018). Thus, we evaluated NLRP3 activation and intrahepatic inflammation in the livers post‐IR. Indeed, the aged group had enhanced expressions of NLRP3, TNF‐α, IL‐6, IL‐1β, IL‐18, MCP‐1, and CXCL‐10 (Figure 1d), accompanied by higher levels of serum IL‐1β, IL‐18, MCP‐1, and CXCL‐10 (Figure 1e). IFN‐β was elevated in both young and aged livers post‐IR. However, no significant differences were observed between the young and aged groups (Figure 1e). Enhanced NLRP3 and Cleaved caspase‐1 activation was observed in the results of the Western blot analysis of the aged livers post‐IR (Figure 1f). These results demonstrated that aging enhanced intrahepatic NLRP3 activation and aggravated liver IR injury.

### 2.2 Aging increased liver IR injury by promoting NLRP3 activation in macrophages

**FIGURE 2:**
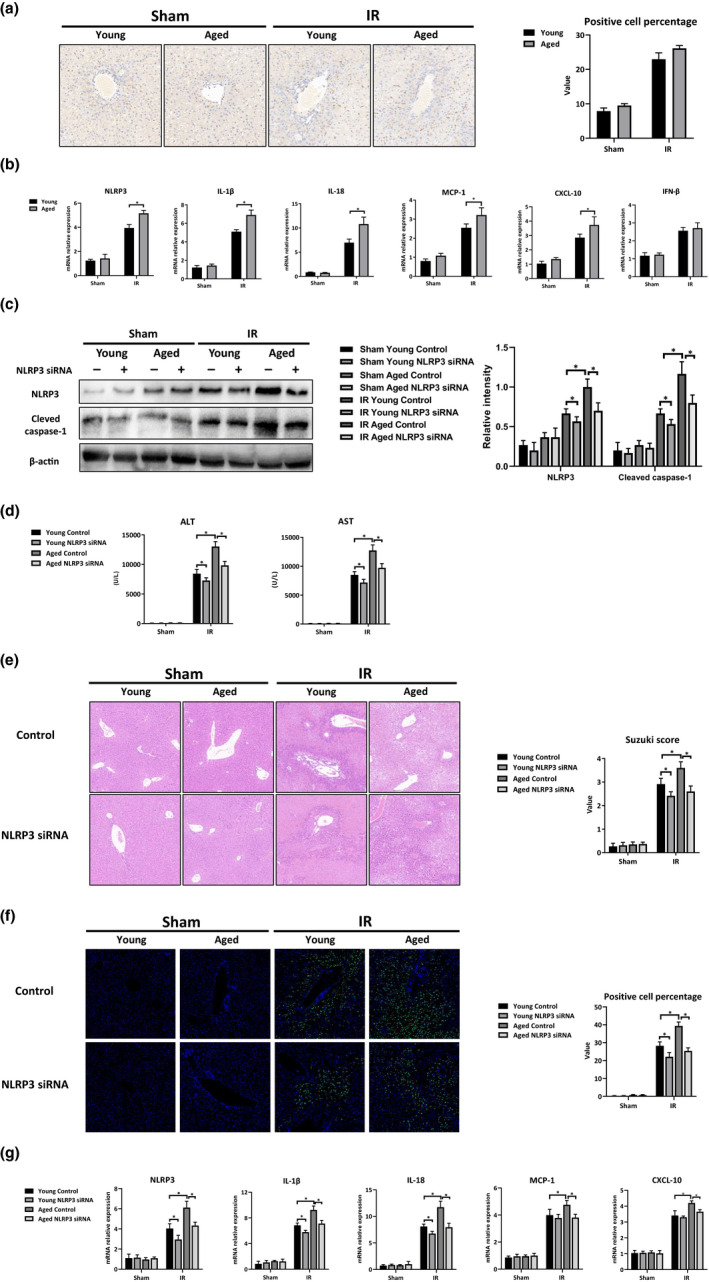
Aging increased liver IR injury by promoting NLRP3 activation in macrophages. Young and aged mice were subjected to liver partial warm ischemia for 1.5 hr followed by 6 hr of reperfusion. KCs were isolated from the livers of each group after operations. (a) IHC F4/80‐stained liver tissue sections; positive cell percentage was measured using ImageJ software. (b) Inflammation‐related gene expressions (NLRP3, IL‐1β, IL‐18, MCP‐1, CXCL‐10, and IFN‐β) of KCs were measured by qRT‐PCR, and the average target gene/GAPDH ratios of different experimental groups were presented. Mice were pretreated with NLRP3 siRNA (5 mg/kg) or non‐specific siRNA (Control) 3 hr before liver partial warm IR. Mice were sacrificed 6 hr after reperfusion. KCs were isolated from the livers after operations. (c) Western blot analysis results of NLRP3, Cleaved caspase‐1, and β‐actin expression of KCs. Relative intensity was analyzed by ImageJ software. (d) Average levels of serum ALT and AST in the mice. (e) H&E‐stained section of livers; average Suzuki scores were based on the H&E‐stained liver sections from different groups of mice. (f) TUNEL assay (green) was used to detect DNA fragmentation in the livers of the young and aged mice, and DAPI (blue) was used as a counterstain. The value of the integrated density/cell was calculated with ImageJ software. (g) NLRP3, IL‐1β, IL‐18, MCP‐1, and CXCL‐10 gene expressions of livers were measured by qRT‐PCR. Average target gene/GAPDH ratios of different experimental groups were shown. n = 6 mice/group. All results were representative of at least two independent experiments. Values were presented as the mean ± *SD*. Significance (*p*‐value) was determined by *t* test, *p < 0.05.

NLRP3 activation in macrophages has been implicated as important in the pathogenesis of liver IR injury (Lu et al., 2016). Therefore, we investigated the role of NLRP3 activation in macrophages that regulate IR injury in the aged livers. As shown in Figure 2a, there was no significant difference in the number of infiltrated macrophages in the livers post‐IR between the young and aged mice. However, macrophages isolated from the aged livers post‐IR showed increased levels of NLRP3 and IL‐1β, IL‐18, MCP‐1, and CXCL‐10 (Figure 2b). IFN‐β was elevated in both young and aged macrophages post‐IR, with no significant differences between the young and aged groups (Figure 2b).

To further determine the role of NLRP3 activation in macrophages in regulating IR injury in aged livers, we used mannose‐conjugated polymers to deliver NLRP3 siRNA or a scramble non‐specific siRNA (Control) specifically to phagocytes in vivo. NLRP3 induction and the subsequent activation of Cleaved caspase‐1 in macrophages in both the young and aged mice post‐IR was effectively inhibited by NLRP3 siRNA but not by the non‐specific siRNA (Figure 2c). Moreover, NLRP3 siRNA administration protected livers against IR injury in both young and aged mice, as shown by the reduced levels of serum ALT and AST, better preserved liver architecture with lower Suzuki scores and fewer TUNEL‐positive stained hepatocytes (Figure 2d‐f, IR: Young NLRP3 siRNA vs. Young Control; Aged NLRP3 siRNA vs. Aged Control). NL RP3 inhibition also decreased proinflammatory gene induction of NLRP3, IL‐1β, and IL‐18 in young and aged mice post‐IR (Figure 2g, IR: Young NLRP3 siRNA vs. Young Control siRNA; Aged NLRP3 siRNA vs. Aged Control). In addition, NLRP3 knockdown reduced MCP‐1 and CXCL‐10 expressions in aged mice, but not in young mice (Figure 2g, IR: Aged NLRP3 siRNA vs. Aged Control; Young NLRP3 siRNA vs. Young Control). Notably, compared with the effects found in the young mice, the protective effects of the NLRP3 blockade on liver IR injury were more significant in the aged mice, leading to comparable liver IR injury and intrahepatic inflammation in both the young and aged mice post‐IR (Figure 2d‐g, IR: Young NLRP3 siRNA vs. Aged NLRP3 siRNA). Furthermore, NLRP3 inhibition in the macrophages decreased neutrophil infiltration and promoted Tregs activation in the both young and aged mice post‐liver IR (Figure S2). Thus, enhanced NLRP3 activation in macrophages was essential for promoting intrahepatic inflammation and exacerbating liver injury in the aged mice post‐IR.

### 2.3 Aging promoted NLRP3 activation in macrophages in a STING‐dependent manner

**FIGURE 3:**
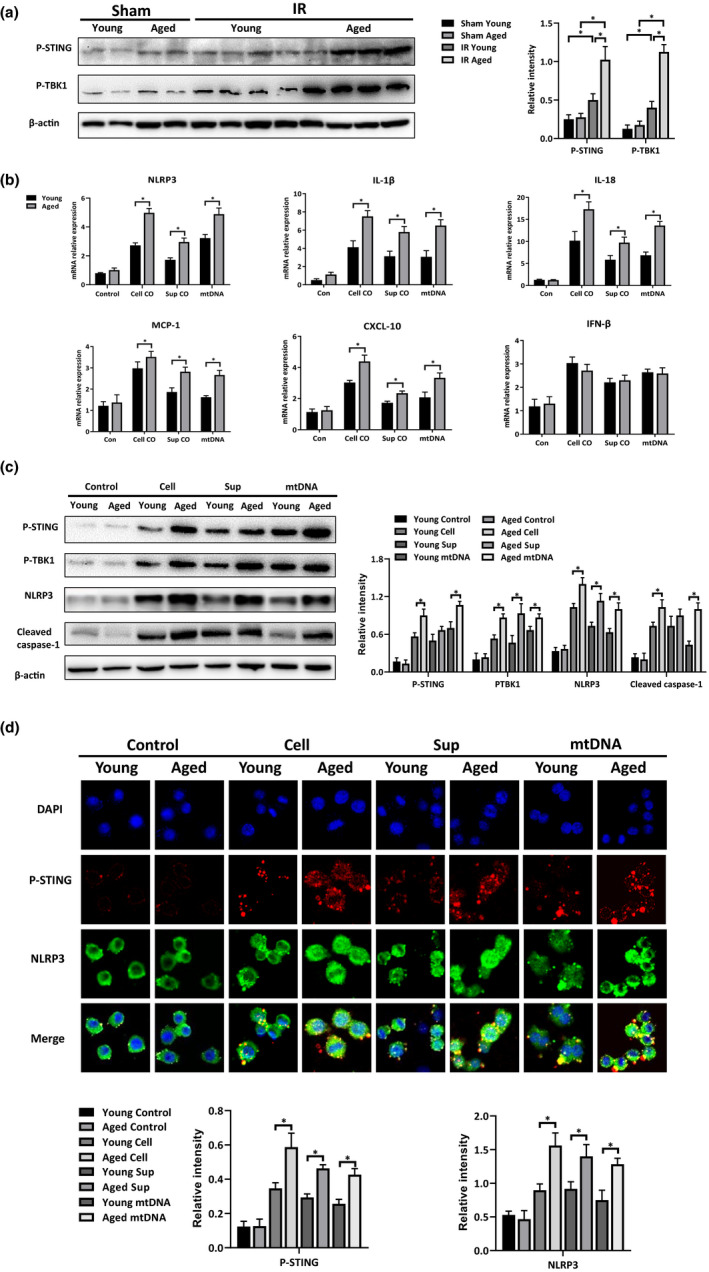
Aging aggravated mtDNA induced‐inflammation through STING activation in macrophages. Mice were subjected to liver partial warm ischemia for 1.5 hr followed by 6 hr of reperfusion. KCs were isolated from the livers of each group. (a) Western blot analysis of P‐STING, P‐TBK1, and β‐actin expression in KCs; relative intensity levels were analyzed by ImageJ software. BMDMs were co‐cultured with HR‐stressed hepatocytes, the supernatant or mtDNA (100 ng/ml) from HR‐stressed primary hepatocytes. BMDMs were harvested 6 hr later, and the cellular proteins and RNA were prepared for further study. (b) NLRP3, IL‐1β, IL‐18, MCP‐1, CXCL‐10, and IFN‐β gene expressions in BMDMs were measured by qRT‐PCR. Average target gene/GAPDH ratios of different experimental groups were presented. (c) Western blot analysis of P‐STING, P‐TBK1, NLRP3, Cleaved caspase‐1, and β‐actin expression in BMDMs; relative intensity levels were evaluated by ImageJ software. (d) Staining of P‐STING (red), NLRP3 (green), and nuclei (DAPI) in BMDMs; the integrated density/cell values were determined by ImageJ software. n = 6 mice/group. All results were representative of at least two independent experiments. Values were presented as the mean ± *SD*. Significance (*p*‐value) was determined by *t* test, *p < 0.05.

STING is a signaling molecule that elicits a powerful type I interferon response and innate immune activation upon stimulation (Motwani, Pesiridis, & Fitzgerald, 2019; Ablasser & Chen, 2019). Recent studies have also revealed the role of STING in regulating macrophage activation in various liver diseases (Zhang et al., 2019; Luo et al., 2018; Yu et al., 2019). Next, we examined whether aging affected STING activation during IR. mtDNA has been recognized as an important endogenous DAMPs, which can be detected by STING‐dependent sensors (White et al., 2014). We measured mtDNA release from IR‐stressed hepatocytes in young and aged mice. Elevated mtDNA release was observed in the aged hepatocytes post‐IR (Figure S1). As shown in Figure 3a (IR Young vs. Sham Young), liver IR induction slightly upregulated STING signaling, as shown by the slightly increased expression of phosphorylated STING Ser365 (P‐STING) and phosphorylated TANK‐binding kinase 1 Ser172 (P‐TBK1) in the macrophages post‐IR. In contrast, the macrophages from aged livers demonstrated significantly increased protein levels of P‐STING and P‐TBK1 post‐IR (Figure 3a, IR Aged vs. Sham Aged).

To further study the role of STING signaling in regulating macrophage activation, BMDMs were isolated from young and aged mice and co‐cultured with hypoxia and reoxygenation (HR)‐stressed primary hepatocytes (Cell) or its supernatant (Sup) or with mtDNA isolated from primary hepatocytes post‐HR (mtDNA). Interestingly, all three treatments triggered STING and NLRP3 activation in the BMDMs from both the young and aged mice, as shown by the results of the Western blot analysis of P‐STING, P‐TBK1, NLRP3, and Cleaved caspase‐1 (Figure 3c), as well as proinflammation‐related gene expressions (Figure 3b). Moreover, compared with those from the young group, the stimulated BMDMs from aged mice showed much higher protein levels of P‐STING, P‐TBK1, NLRP3, and Cleaved caspase‐1 as measured by Western blot analysis (Figure 3c) and immunofluorescence assay (Figure 3d).

**FIGURE 4:**
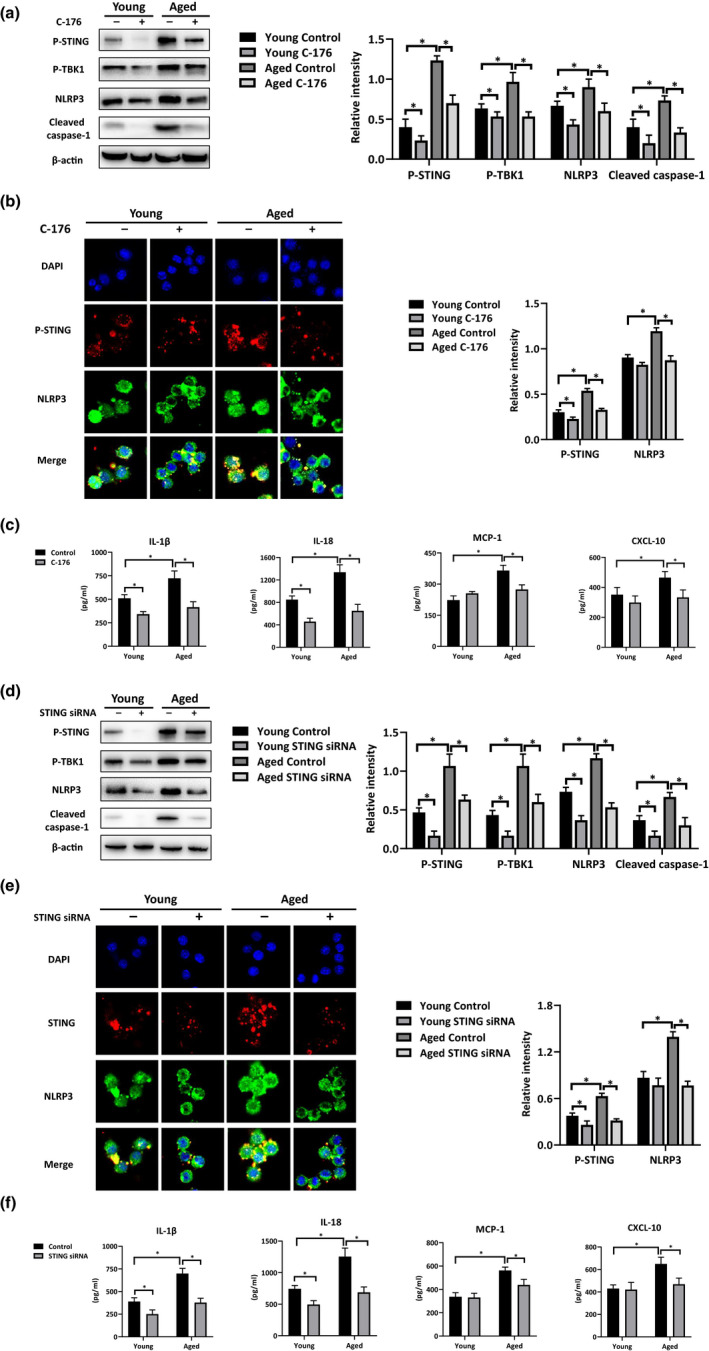
Aging aggravated mtDNA induced‐inflammation through STING‐dependent NLRP3 activation in macrophages. BMDMs were pretreated with C‐176 (20 µM) or a vehicle control 3 hr before mtDNA stimulation (100 ng/ml). The cells and culture supernatant were collected 6 hr after stimulation. (a) Treated BMDMs were analyzed for P‐STING, P‐TBK1, NLRP3, and Cleaved caspase‐1 levels by Western blotting; relative levels of protein expression were evaluated by ImageJ software. (b) Staining of P‐STING (red), NLRP3 (green), and nuclei (DAPI) in BMDMs; the integrated density/cell values were determined by ImageJ software. (c) The levels of cytokines/chemokines (IL‐1β, IL‐18, MCP‐1, and CXCL‐10) in the cell culture supernatant were measured by ELISA. BMDMs were pretreated with STING siRNA (10 µM) or non‐specific siRNA (Control) 48 hr before mtDNA stimulation (100 ng/ml). The cells and culture supernatant were collected 6 hr after stimulation. (d) Treated BMDMs were analyzed for the levels of P‐STING, P‐TBK1, NLRP3, and Cleaved caspase‐1 by Western blotting; relative levels of protein expressions were evaluated by ImageJ software. (e) Staining of P‐STING (red), NLRP3 (green), and nuclei (DAPI) in BMDMs; the value of integrated density/cell determined by ImageJ software. (f). The levels of cytokines/chemokines (IL‐1β, IL‐18, MCP‐1, and CXCL‐10) in the cell culture supernatant were measured by ELISA. All results were representative of at least two independent experiments. Values were presented as the mean ± *SD*. Significance (*p*‐value) was determined by *t* test, *p < 0.05.

To determine the importance of STING in NLRP3 regulation by aging in macrophages, C‐176, a specific inhibitor of STING, and STING siRNA were used to block STING activation. The BMDMs from the young and aged mice were pretreated with C‐176 or STING siRNA followed by stimulation with mtDNA. The C‐176 treatment effectively inhibited STING activation in both young and aged BMDMs post‐mtDNA stimulation, as shown by the decreased protein levels of P‐STING and P‐TBK1 (Figure 4a). More importantly, STING inhibition by C‐176 blocked the over‐activation of NLRP3 signaling in the BMDMs from the young and aged mice post‐mtDNA stimulation, as shown by decreased levels of NLRP3 and Cleaved caspase‐1 expression (Figure 4a), findings that were confirmed by immunofluorescent staining of STING and NLRP3 (Figure 4b). Functionally, the increased level of proinflammatory cytokines/chemokines secretion were abrogated by STING inhibition with C‐176 in the mtDNA‐stimulated BMDMs of young and aged mice, as shown by significantly decreased levels of IL‐1β, IL‐18, MCP‐1, and CXCL‐10 in supernatant medium (Figure 4c). To avoid the non‐specific and off‐target effects of the small‐molecule inhibitor, STING siRNA was also used for STING inhibition. Similar results were observed (Figure 4d‐f). These results suggested that STING was required for NLRP3 over‐activation in the macrophages from aged mice.

### 2.4 Aging aggravated IR injury by promoting STING‐dependent NLRP3 activation in macrophages

**FIGURE 5:**
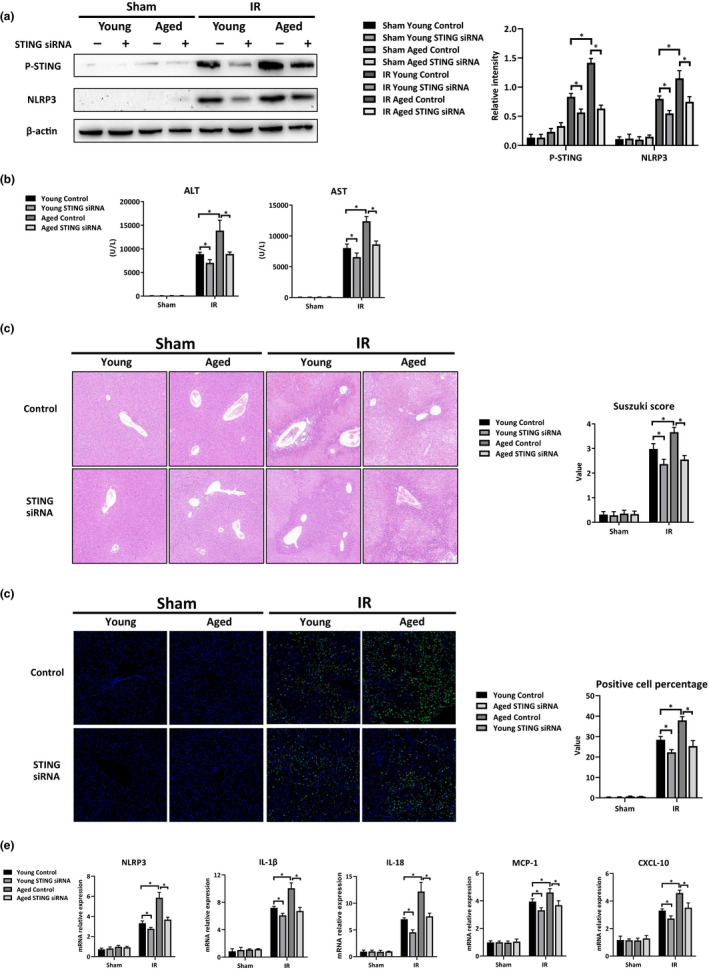
Aging aggravated IR injury by promoting STING‐dependent NLRP3 activation in macrophages. Mice were pretreated with STING siRNA (5 mg/kg) or non‐specific siRNA (Control) 3 hr before liver partial warm IR. Mice were sacrificed 6 hr after reperfusion. KCs were isolated from livers of each group. (a) Western blot analysis of P‐STING, P‐TBK1, and β‐actin expression of KCs. Relative intensity analyzed by ImageJ software. (b) Average serum ALT and AST levels in mice. (c) H&E‐stained liver tissue sections from each group; average Suzuki scores were based on the H&E‐stained liver tissue sections. (d) TUNEL staining of liver tissue sections; positive cell percentage was measured by ImageJ software. (e) NLRP3, IL‐1β, IL‐18, MCP‐1, and CXCL‐10 gene expressions in livers were measured by qRT‐PCR. Average target gene/GAPDH ratios of different experimental groups were presented. n = 6 mice/group. All results were representative of at least two independent experiments. Values were presented as the mean ± *SD*. Significance (*p*‐value) was determined by *t* test, *p < 0.05.

To further dissect the effects of STING‐dependent NLRP3 activation of macrophages during IR injury in aged mice, mannose‐conjugated polymers with STING siRNA were used to knock down STING activation in macrophages in vivo (Figure 5a). STING siRNA administration protected the livers against IR injury in both the young and aged mice, as shown by the reduced levels of serum ALT and AST (Figure 5b), better preserved liver architecture with lower Suzuki scores (Figure 5c), fewer TUNEL‐positive stained hepatocytes (Figure 5d) and decreased induction of intrahepatic proinflammation‐related genes (Figure 5e). STING inhibition in the macrophages abrogated the detrimental role of aging in aggravating liver injury and intrahepatic inflammation in the livers post‐IR (Figure 5a‐e, IR: Young STING siRNA vs. Aged STING siRNA). Interestingly, STING inhibition also reduced mtDNA release (Figure S1), decreased neutrophil infiltration, and promoted Treg activation in aged mice post‐IR (Figure S2).

### 2.5 Aging promoted NLRP3 activation in humans post‐IR

**FIGURE 6:**
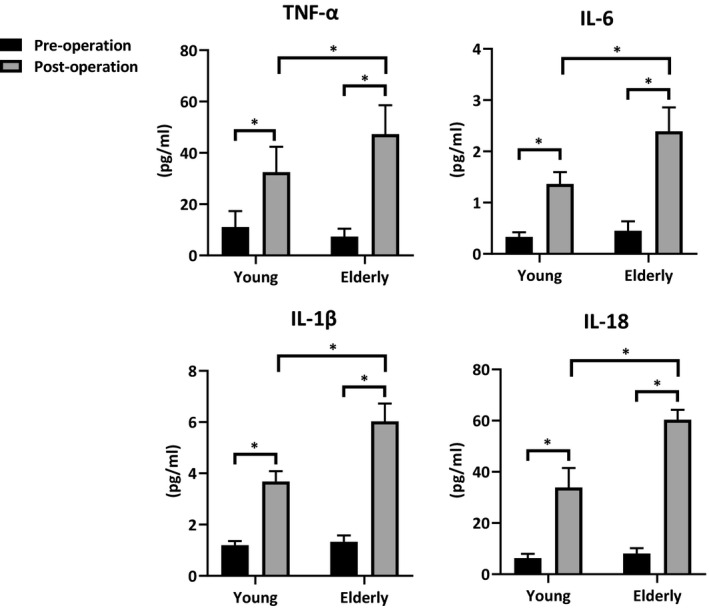
Aging promoted NLRP3 activation in humans post‐IR. Peripheral blood was collected respectively before transplantation and 12 hr post‐operation for study. TNF‐α, IL‐6, IL‐1β, and IL‐18 levels in patients' serum were measured by ELISA. All results were representative of at least two independent experiments. Values were presented as the mean ± *SD*. Significance (*p*‐value) was determined by *t* test, *p < 0.05.

Finally, to evaluate the clinical relevance of NLRP3 signaling regulated by aging during liver ischemia, we collected human peripheral blood from young and elderly patients undergoing liver transplantation. The levels of NLRP3 activation and inflammation were analyzed by ELISA. As shown in Figure 6 (Post‐operation vs. Pre‐operation, Young/Elderly), significantly increased levels of serum TNF‐α, IL‐6, IL‐1β, and IL‐18 were found in the patients post‐IR stress by transplantation. Moreover, the elderly patients showed much higher levels of these inflammatory cytokines and chemokines post‐transplantation (Figure 6, post‐operation: Elderly vs. Young). All patients recovered well, no significant difference was observed in regarding the time of post‐operative hospital stay, and none patients occurred liver failure or acute rejection. These findings confirmed that aging promoted excessive inflammation and NLRP3 over‐activation during liver IR injury.

## 3 DISCUSSION

Although we found liver IR injury to be aggravated in aged mice in our previous study (Jiang et al., 2019), which was consistent with findings from other studies (Okaya et al., 2005; Selzner et al., 2009), the underlying mechanism remains to be determined. In the present study, we demonstrated that aging aggravated IR injury by promoting STING‐dependent NLRP3 activation in macrophages, which provided a novel regulatory mechanism of macrophage innate immune activation in aged mice during IR injury.

Multiple alterations at the cellular and molecular levels contributed to the increased liver injury post‐IR in the aged mice, among which the enhanced inflammatory response has been shown to be an important factor (Kan et al., 2018). Age‐dependent loss of induced regulatory T‐cell function has also been shown to exacerbate liver IR injury in a recent study (Liu et al., 2018). However, controversial results have been found regarding the proinflammatory response of macrophages from young and aged subjects. Fagiolo U measured in vitro cytokines production of peripheral mononuclear cells from healthy young and elderly people and found significantly increased levels of IL‐6, TNF‐α, and IL‐1β but similar levels of IFN‐γ in the mitogen‐stimulated cultured cells from the elderly donors (Fagiolo et al., 1993). Sadeghi, Schnelle, Thoma, Nishanian, and Fahey (1999) found that monocyte‐derived macrophages from elderly persons produced higher levels of IL‐1β and IL‐6 at a steady state but lower levels of IL‐1β and higher levels of IL‐6 and IL‐10 secretion upon stimulation. In another study, monocyte‐derived macrophages from aged and young individuals had similar levels of TNF‐α, IL‐6, IL‐1β, and MCP‐1 release in vitro at a steady state and upon LPS stimulation (Seidler, Zimmermann, Bartneck, Trautwein, & Tacke, 2010). In the present study, we found that macrophages from the livers of the aged mice secreted higher levels of IL‐1β, IL‐18, MCP‐1, and CXCL‐10 post‐IR in vivo and post‐mtDNA stimulation in vitro.

Critical roles for NLRP3 have been found in the regulation of liver IR injury (Xu et al., 2018). Proinflammatory mediators such as ROS and high mobility group box 1 (HMGB1) produced during IR injury to the liver could activate NLRP3. Gene silencing of NLRP3 protected livers against IR injury in mice (Zhu et al., 2011). Furthermore, Kim HY et al. found that depletion of KCs markedly decreased NLRP3 and AIM2 inflammasome activation, indicating that activation of NLRP3 and AIM2 inflammasomes in KCs contributed to the pathogenesis of hepatic IR injury (Kim et al., 2015). Endogenous extracellular histones activated the NLRP3 inflammasome in macrophages through TLR9, which triggered sterile inflammation during liver IR injury (Huang et al., 2013). A recent study also reported that NLRP3 activation in macrophages was controlled by the HSF1‐β‐catenin axis and promoted liver IR injury in mice (Yue et al., 2016).

Emerging evidence of aging‐related NLRP3 activation has been reported. Spontaneously elevated TNF levels were observed in aged mice and were found to be critical for increased NLRP3 expression and caspase‐1 activity in adipose and liver tissues (Bauernfeind et al., 2016). Ablation of the NLRP3 inflammasome protected mice from aging‐related increases in innate immune activation and systemic low‐grade aging‐related sterile inflammation (Youm et al., 2013). Bone marrow‐derived and alveolar macrophages from aged mice had higher levels of NLRP3 inflammasome activation and caspase‐1–dependent IL‐1β and IL‐18 production, which contributed to the development of experimental pulmonary fibrosis (Stout‐Delgado et al., 2016). In the present study, we found that NLRP3 was activated in both young and aged mice and that NLRP3 activation was enhanced in aged mice post‐IR. Furthermore, inhibition of NLRP3 abrogated the increase in liver IR injury in the aged mice compared with increase in the young mice. Thus, enhanced NLRP3 activation in macrophages may contribute to the development of aggravated liver IR injury in aged mice.

STING, a protein with 379 amino acids, is expressed in various cell types and has been shown to play multiple critical roles in regulating infection and inflammation (Barber, 2015). Early studies revealed that STING was essential for the immune response to bacteria and virus invasion. Recent studies have also found that STING signaling could also be activated by self‐DNA in necrotic cells, which subsequently initiated autoinflammatory diseases. Specifically, cytosolic DNA species could bind to cyclic GMP–AMP synthase (cGAS), leading to the production of a type of cyclic dinucleotide (CDN). After binding to these CDNs, STING forms a complex with TBK1 to induce signaling transduction and ultimately to IRF3 and NF‐kB activation.

Increasing evidence has been reported regarding the regulatory role of STING signaling in various liver diseases. Due to the lack of STING expression, human and murine hepatocytes did not produce type I IFN in response to HBV infection. However, introduction of STING expression in these hepatocytes reconstituted the STING signaling pathway, leading to improved HBV control (Thomsen et al., 2016). Blocking STING signaling has been identified as an important mechanism for HCV evasion of host innate immunity (Ding et al., 2013). Luo et al. (2018) found that liver tissues from patients with non‐alcoholic fatty liver disease and mice with HFD‐induced steatosis expressed higher levels of STING, while STING inhibition in macrophages decreased the inflammation and the severity of the liver fibrosis. The mtDNA from hepatocytes of HFD‐fed mice induced TNF‐α and IL‐6 expression in KCs, which was inhibited when STING was inhibited (Yu et al., 2019).

Few studies have shown the role of STING signaling in mediating inflammation in aging‐related conditions. Cells from older subjects harbored higher levels of extranuclear DNA than cells from younger subjects, which triggered innate immune responses through the DNA‐sensing cGAS‐STING pathway (Lan et al., 2019). Lutz Hamann et al. investigated the influence of a STING mutant, which led to known impaired function and found that STING SNP R293Q was associated with a decreased risk of aging‐related diseases (Hamann et al., 2019). Here, we demonstrated that elevated mtDNA release from the aged hepatocytes post‐IR, which was suppressed by STING inhibition in macrophages. These findings indicated that the elevated mtDNA in aged mice post‐IR may be at least partially caused by enhanced macrophage proinflammatory activation. Other factors such as the energy metabolism and autophagy may directly affect the hepatocellular cell injury and mtDNA release as well (Niazi, Schneekloth, & Taner, 2017).

Recent studies have shown that mtDNA released in the cytoplasm played a key role in promoting NLPR3 inflammasome activation (Shimada et al., 2012; Nakahira et al., 2011). Li, Zhou, et al. (2019) found that LPS stimulation triggered perinuclear STING translocation and interferon regulatory Factor 3 (IRF3) phosphorylation, leading to subsequent NLRP3 activation, which contributed to cardiac dysfunction and inflammation. In the present study, we found that mtDNA stimulation triggered STING and NLRP3 activation in macrophages and that inhibition of STING signaling decreased NLRP3 expression in the macrophages of aged mice.

A major limitation of our study is the lack of STING deficiency mice. Although the siRNA or inhibitor was able to effectively inhibit STING activation, it may have off‐target and cytotoxic side effects. Thus, the use of STING deficiency mice would provide more powerful evidence to support our conclusion and will be critical for our future studies.

## 4 SUMMARY

In summary, this is the first study to suggest an important role for the STING‐NLRP3 pathway in regulating macrophage innate immune activation and enhanced liver IR injury in aged mice. Therefore, targeting STING to inhibit macrophage excessive proinflammatory activation in macrophages would be a viable therapeutic or preventive approach for the management of aggravated liver IR injury in aged patients.

## 5 EXPERIMENTAL PROCEDURES

### 5.1 Animals

Young (8 weeks) and aged (100 weeks) male C57/BL6 mice were purchased from GemPharmatech Co., Ltd. The mice were housed and maintained under a 12 hr light/dark cycle with ad libitum access to water and standard chow with supplements under specific pathogen‐free conditions. All animal work was performed according to the “Guide for the Care and Use of Laboratory Animals” published by the National Research Council.

### 5.2 Liver IR injury model

A model of partial hepatic warm IR injury was used as described previously (Zhou et al., 2018). In brief, after successful anesthesia with 2.5% isoflurane, the mice were injected intraperitoneally with heparin (100 mg/kg). An atraumatic clip was used to interrupt the arterial and portal venous blood supply to the cephalad lobes of the liver. After 90 min of ischemia, the clip was removed, initiating hepatic reperfusion. Sham controls underwent the same procedure but without vascular occlusion. The mice were sacrificed after 6 hr of reperfusion.

### 5.3 Serum biochemical measurements and liver histopathology

Serum ALT and AST levels were measured with an AU680 clinical chemistry analyser (Beckman Coulter). Some liver specimens were fixed in 4% paraformaldehyde and embedded in paraffin. Liver sections were stained with H&E or F4/80. The severity of liver the ischemia/reperfusion injury was graded using Suzuki score. Tissues without necrosis or congestion/centrilobular ballooning were given a score of 0, whereas those presenting with severe congestion and/or >60% lobular necrosis were given a score of 4.

### 5.4 Isolation and treatment of liver cells

Livers were perfused in situ via the portal vein with Hanks balanced salt solution (HBSS; Gibco) supplemented with 5% heat‐inactivated FBS, followed by 0.3% collagenase IV (Sigma‐Aldrich). Perfused livers were dissected and teased through 70 µm nylon mesh cell strainers (Corning). Liver cells were suspended and centrifuged at 50 *g* for 2 min for 3 times.

1. The supernatant was collected by centrifugation at 800 *g* for 5 min. Thereafter, cells were suspended and allowed to attach to cell culture plates for 15 min at 37 C, and the attached cells were KCs.
2. Primary hepatocytes were pelleted after centrifugation at 50 *g* for 2 min. Cells were resuspended in 20 ml of 40% cold Percoll solution (Sigma‐Aldrich) and centrifuged at 150 *g* for 7 min. The pelleted hepatocytes were suspended in plating medium (Williams E medium with hepatocyte thawing and plating supplement pack; Gibco) and plated in collagen type I‐coated plates for 3 hr. Maintenance medium (Williams E medium with hepatocyte maintenance supplement pack; Gibco) was used for cultures overnight or longer.

Hepatocytes culture HR patterns were imposed following a method described previously (Strey et al., 2010).

### 5.5 Culture of BMDMs

BMDMs were generated as previously described (Zhou et al., 2018). In brief, bone marrow cells were isolated from femurs and tibias of young and aged mice. The cells were cultured in DMEM supplemented with 10% fetal bovine serum and 20% L929‐conditioned medium for 7 days. The BMDMs were replated and cultured overnight for further experiments.

BMDM stimulation and activation studies: the hepatocytes were subjected to the HR model for 12 hr, the hepatocytes and supernatant were collected, and the mtDNA was extracted from the HR‐stressed hepatocytes using a mitochondrial DNA isolation kit following the instructions (ab65321; Abcam). After incubation with the above hepatocytes (BMDM/hepatocyte at a ratio of 2:1), supernatant or mtDNA (100 ng/ml) for 6 hr, the BMDMs and supernatant were harvested for further analysis.

### 5.6 NLRP3 and STING signaling inhibition

In vivo studies, NLRP3 siRNA or STING siRNA was mixed with mannose‐conjugated polymers (Polyplus Transfection) in a ratio specified by the manufacturer and administered intraperitoneally (siRNA 5 mg/kg; Santa Cruz Biotechnology) 3 hr before the onset of liver ischemia.

In vitro studies, the BMDMs were treated with STING inhibitor C‐176 (20 μM; MedChemExpress, Monmouth Junction, New Jersey, USA)/vehicle control for 3 hr or transiently transfected with STING siRNA (10 μM; Santa Cruz Biotechnology)/non‐specific siRNA using Lipofectamine 3000 (Thermo Fisher Scientific) for 48 hr before mtDNA (100 ng/ml) stimulation. Culture supernatant was collected 6 hr after stimulation to measure cytokines/chemokines levels. The cells were collected 6 hr after stimulation and used for Western blot or qRT‐PCR analysis.

### 5.7 Quantitative reverse transcription PCR

Total RNA (2.0 mg) was reverse transcribed into complementary DNA using an RR047A PrimeScript RT reagent kit with gDNA Eraser (TaKaRa). qRT‐PCR was performed with a StepOnePlus Real‐Time PCR system (Thermo Fisher Scientific, Waltham, Massachusetts, USA) in a final reaction volume of 20 μl, containing 1× TB Green Premix (TaKaRa), complementary DNA, and each primer at 0.125 μM. The amplification conditions were as follows: 50°C for 2 min, 95°C for 10 min followed by 40 cycles of 95°C for 15 s, and 60°C for 1 min.

### 5.8 Western blotting

Tissues and cellular proteins were extracted with ice‐cold RIPA lysis buffer (Beyotime, Shanghai, China) supplemented with protease and phosphatase inhibitors (Beyotime, Shanghai, China). Protein concentrations were determined by a Bradford BCA assay (Beyotime, Shanghai, China). Proteins (30 μg) were subjected to 10% SDS‐PAGE electrophoresis and transferred to a Polyvinylidene Fluoride (PVDF) nitrocellulose membrane. Antibodies against P‐STING, P‐TBK1, NLRP3, Cleaved caspase‐1, and β‐actin (Cell Signaling Technology) were used and incubated overnight at 4 °C. After 2 hr of incubation with the appropriate HRP‐conjugated secondary antibody, bands were detected with Immobilon ECL Ultra Western HRP substrate (Millipore), and images were taken using a Tanon chemiluminescent imaging system (Tanon). Densitometry to determine changes in protein expression was measured using ImageJ software.

### 5.9 ELISA

The secretion of cytokines/chemokines (TNF‐α, IL‐1β, IL‐6, IL‐18, MCP‐1, CXCL‐10) was measured by ELISA, according to the manufacturer's protocols (Thermo Fisher Scientific).

### 5.10 Detection of mtDNA in cytosolic extracts

The method of mtDNA detection in cytosolic extracts followed the protocol described previously (West et al., 2015). In brief, primary hepatocytes were subjected to HR. The whole cell extracts served normalization controls for total mtDNA. Cytosolic fractions were isolated by centrifugation as described previously (West et al., 2015). DNA was then isolated from whole cell extracts and cytosolic fractions using Qiaquick nucleotide removal columns (Qiagen). qRT‐PCR was performed on both whole cell extracts and cytosolic fractions.

### 5.11 Confocal microscopy

The samples for confocal immunofluorescent staining were stored at −80°C for frozen sectioning. The frozen sections were cut into 4 μm slices, blocked and permeated with 3% BSA‐0.5% Triton for 30 min at room temperature, and incubated with primary antibody to detect P‐STING or NLRP3 (Abcam, Cambridge, England) at 4°C overnight. Donkey anti‐rabbit IgG H&L (Alexa Fluor 647; Abcam) or donkey anti‐goat IgG H&L (Alexa Fluor 488; Abcam) was used to visualize the primary antibody. Nuclei were stained with 4′, 6‐diamidino‐2‐phenylindole dihydrochloride (DAPI, Invitrogen) Images were captured and analyzed with a confocal microscope (Carl Zeiss).

### 5.12 Patients and specimens

A total of 12 patients (6 young and 6 elderly men) with hepatocellular carcinoma and undergoing liver transplantation were included in the current study. Patients aged over 65 were considered as elderly group (Eufrasio et al., 2011{Niazi, 2017 #39)}. The mean age (±*SD*) of the young group was 33 ± 4.76 years, while that elderly group was 70.17 ± 2.92 years. Peripheral blood was collected respectively before transplantation and 12 hr post‐operation for study. The study protocol was approved by the Institutional Review Board of The First Affiliated Hospital with Nanjing Medical University (Institutional Review Board approval number 2017SRFA‐138). Informed consent was obtained from each patient.

### 5.13 Data analysis

All results were representative of at least two independent experiments. Results were shown as the mean ± standard deviation (*SD*). Multiple group comparisons were performed by one‐way analysis of variance followed by Bonferroni's post hoc test. All analyses were performed with Graphpad8.0. *p*‐value < 0.05 (two‐tailed) was considered statistically significant.

## CONFLICT OF INTEREST

The authors disclosed no conflicts of interest.

## AUTHOR CONTRIBUTIONS

WZ, HZ, and XW conceived the project, designed experimental strategies, and drafted and revised the manuscript for publication, WZ, ZR, and JR performed the experiments and did data analysis. GH, PW, TJ, XP, and SZ collected clinical samples and assisted analysis. XW, HZ, and ZR provided funding support and supervised the study.

## Supporting information

Figures S1‐S2

## Data Availability

All data supporting the findings of this study are available within the paper.
