# Supplementary figures and images for: Aging aggravated liver ischemia and reperfusion injury by promoting STING‐mediated NLRP3 activation in macrophages
Source: Aging Cell. 2020 Jul 14;19(8):e13186. doi: 10.1111/acel.13186 (PMC7431827; doi:10.1111/acel.13186)

Fig.S1

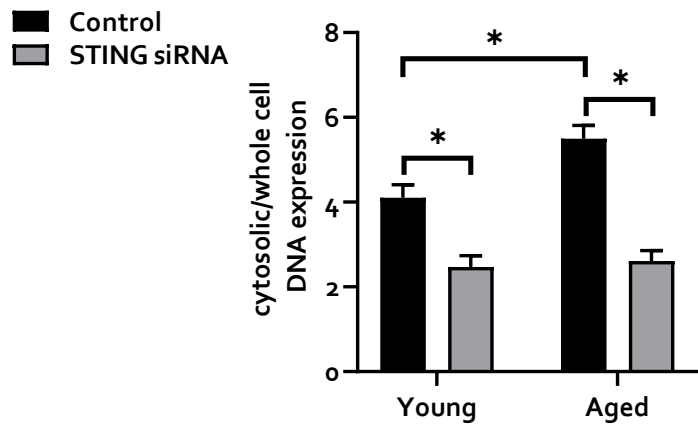

Fig.S2

a

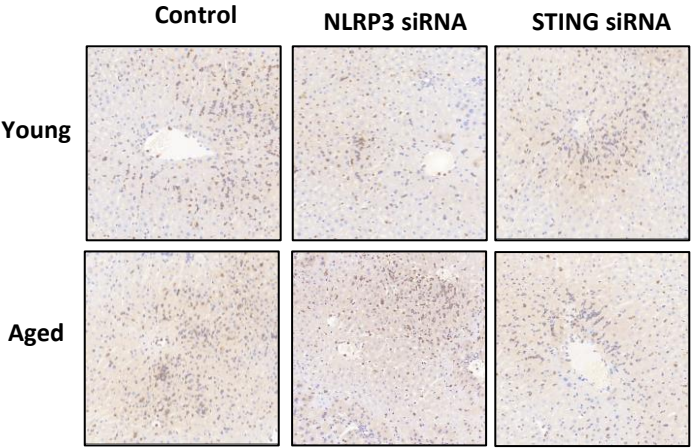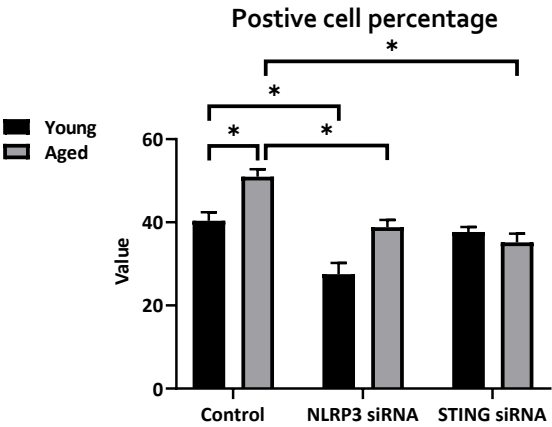

b

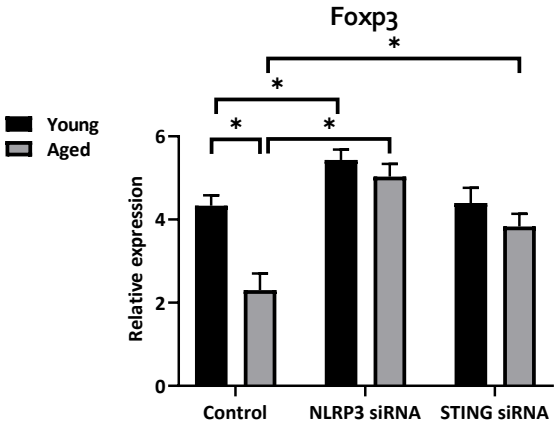

Supplement: Supplementary file 1 — Figures S1‐S2 [file ACEL-19-e13186-s001.pdf]
